# The Reporting Quality of Systematic Reviews and Meta-Analyses in Industrial and Organizational Psychology: A Systematic Review

**DOI:** 10.3389/fpsyg.2017.01395

**Published:** 2017-08-22

**Authors:** Naomi Schalken, Charlotte Rietbergen

**Affiliations:** Department of Methodology and Statistics, Faculty of Social and Behavioral Sciences, Utrecht University Utrecht, Netherlands

**Keywords:** systematic review, reporting quality, transparency, industrial and organizational psychology, MARS, replicability

## Abstract

**Objective:** The goal of this systematic review was to examine the reporting quality of the method section of quantitative systematic reviews and meta-analyses from 2009 to 2016 in the field of industrial and organizational psychology with the help of the Meta-Analysis Reporting Standards (MARS), and to update previous research, such as the study of Aytug et al. (2012) and Dieckmann et al. (2009).

**Methods:** A systematic search for quantitative systematic reviews and meta-analyses was conducted in the top 10 journals in the field of industrial and organizational psychology between January 2009 and April 2016. Data were extracted on study characteristics and items of the method section of MARS. A cross-classified multilevel model was analyzed, to test whether publication year and journal impact factor (JIF) were associated with the reporting quality scores of articles.

**Results:** Compliance with MARS in the method section was generally inadequate in the random sample of 120 articles. Variation existed in the reporting of items. There were no significant effects of publication year and journal impact factor (JIF) on the reporting quality scores of articles.

**Conclusions:** The reporting quality in the method section of systematic reviews and meta-analyses was still insufficient, therefore we recommend researchers to improve the reporting in their articles by using reporting standards like MARS.

## Introduction

Because systematic reviews and meta-analyses are often used as evidence-base by clinicians, government officials and other professionals, it is of great importance that the methodological quality of these reviews is high. According to Davis et al. (2014) in their study of practical issues in systematic reviews and meta-analyses in social research, a systematic review is a technique to systematically search, select and evaluate research for a specific research question and to synthesize the reported results. The collection of statistical techniques to synthesize the quantitative results of individual studies, is called meta-analysis. The importance of high methodological quality is for example illustrated by Valentine et al. (2010) in their review of the effects of after-school programs, where evaluated syntheses varied widely in their methods and rigor, and therefore led to differences in conclusions and implications. As a consequence, policymakers could make different decisions based on these varying results. The potential influence of methodological decisions on the final results and conclusions of meta-analyses was discussed by several researchers, see studies 1–5 in Table 1.

**Table 1:** Summary of previous studies on methodological decisions and/or reporting quality.

	**Author(s)**	**Topic**	**Research methodology**	**Findings**
**STUDIES ON METHODOLOGICAL DECISIONS**				
1	Wanous et al., 1989	The influence of judgment calls on the results of meta-analyses in the field of I/O psychology	The consequences of judgment calls on the final results of four pairs of meta-analyses were analyzed	Judgment calls made during the research process resulted in differences in results in the pairs of meta-analyses. These differences were mainly caused by judgment calls in the definition of inclusion criteria, in the data extraction, in the search and in the selection of studies. Interpretation of authors could also play a role in differences in the final results
2	Ada et al., 2012	The influence of methodological decisions on the final conclusions of meta-analyses about the business value of information technology	Several hypotheses were tested while varying the meta-analytic decisions such as the inclusion of studies, the exclusion of outliers and the selection of the statistical meta-analysis method	Meta-analytic choices influenced the results of meta-analyses, especially the choice for inclusion and exclusion criteria and in some cases the choice for the statistical method. Hypotheses with a theoretical foundation were more robust to different decisions made in the meta-analytic process
3	Geyskens et al., 2009	The current state of meta-analytic research in the field of management and the influence of decisions on the final conclusions of meta-analyses	69 meta-analyses between 1984 and 2007 from 14 management journals were evaluated on the researcher decisions made. Also four meta-analyses were performed to investigate the influence of researcher decisions	Decisions regarding the statistical methods in meta-analyses could have substantial influence on the final results of meta-analyses. Important information about choices in statistical methods was omitted in a substantial part of the studied meta-analyses
4	Aguinis et al., 2011	The effects of meta-analytic choices and judgment calls on effect sizes and substantive conclusions of meta-analyses in the field of I/O psychology and management	The content of 196 meta-analyses from 1982 to 2009 including their effect sizes, were analyzed on different methodological choices and judgment calls	Methodological choices and judgment calls had little impact on the final derived effect sizes and substantial conclusions in the meta-analyses
5	Nieminen et al., 2011	The influence of researcher decisions on the processes and findings of meta-analyses about telecommuting	The influence of researcher decisions was studied in three telecommuting meta-analyses	No direct influence of researcher decisions was found on the findings of the meta-analyses, but some differences existed in the prior decisions of meta-analyses, such as in the inclusion criteria and in the selection of studies and moderator variables
**STUDIES ON REPORTING QUALITY**				
6	Aytug et al., 2012	Evaluating the reporting transparency and completeness of meta-analyses in the field of I/O psychology in a systematic review	Meta-analyses from 1995 to 2008 were retrospectively assessed with items of reporting guidelines such as QUOROM and MARS	Only half of the included meta-analyses reported enough information for replication or the assessment of validity. The reporting of the literature search, the inclusion/exclusion criteria and statistical information was often incomplete. Information about limitations of the review, possible bias in primary studies and the amount of heterogeneity was often lacking
7	Kepes et al., 2013	Reviewing the MARS guideline and discussing best practices in meta-analytic reviews in the organizational sciences	Discussion of several MARS items and the implementation of best practices in meta-analyses in the organizational sciences	Meta-analytic reviews did not comply with recommendations in guidelines such as MARS, and still had problems with the accuracy, transparency and replicability of their results
8	Ahn et al., 2012	Reviewing the methodological quality of meta-analyses in the field of education	56 educational meta-analyses from the 2000s were reviewed on different methodological aspects	Meta-analytic practices were adequate in the problem formulation and data collection stages. Improvements could be made in the data evaluation and data analysis stages
9	Dieckmann et al., 2009	Evaluating practice and reporting at each stage of the meta-analytic process	A random sample of meta-analyses from psychology and related fields from 1994 to 2004 were assessed	Improvements could be made in the discussion of publication bias, in the coding procedures and reliability, in dispersion measures, and in the discussion of limitations. Inclusion criteria, the list of primary reports and literature search methods were often fully reported
10	Fehrmann and Thomas, 2011	Evaluating the reporting of computer searches in systematic reviews	Systematic reviews from the PsycINFO and Cochrane Library were evaluated with the Computer Search Report Checklist	The majority of the reviews reported if more than one computer source was used and reported the used alternate search terms. Techniques like truncation searching, the use of a controlled vocabulary, and search tools for articles that cited a study of interest were underreported

In order to judge the methodological quality of systematic reviews and meta-analyses, the reporting quality has to be sufficient (Valentine et al., 2010). Especially when methodological decisions could have an influence on the final results of meta-analyses, extensive reporting of the methodology section is crucial. For example reviewers should describe their extensive search for published and unpublished studies, such as searched databases or whether efforts were made to contact the authors, to judge the risk of publication bias (Wanous et al., 1989; Dieckmann et al., 2009; Valentine et al., 2010; Fehrmann and Thomas, 2011). Furthermore, reviewers should assess the quality of the included studies and report their findings (Valentine et al., 2010). To minimize the risk of bias in the stages of selection, data extraction and quality assessment it is crucial that these steps are executed by at least two researchers (Wanous et al., 1989; Dieckmann et al., 2009; Villasís-Keever and Rendón-Marcías, 2015). In order to judge if there might be bias in one of these stages, review authors should report about the efforts made to prevent this bias.

Reporting has to be done in an objective and neutral way (Villasís-Keever and Rendón-Marcías, 2015) to increase the transparency and replicability of the systematic review or meta-analysis (Kepes et al., 2013). To improve quality, there are guidelines for the reporting of systematic reviews and meta-analyses. The first such guideline was the Quality of Reporting of Meta-analyses statement or QUOROM statement (Moher et al., 1999), designed for meta-analyses of randomized controlled trials. The revision of the QUOROM statement became the Preferred Reporting Items for Systematic reviews and Meta-analyses checklist, the PRISMA statement (Moher et al., 2009). In contrast to the QUOROM checklist the PRISMA checklist was also applicable to other types of reviews than meta-analyses of randomized controlled trials. Following the QUOROM and PRISMA statements that originated from the medical sciences, the American Psychological Association Publications and Communications Board Working Group on Journal Article Reporting Standards (2008) developed a guideline for meta-analyses in psychology, the Meta-Analysis Reporting Standards (MARS). MARS was based on elements from four existing reporting standards, including QUOROM, PRISMA, MOOSE (Meta-analysis of Observational Studies in Epidemiology; Stroup et al., 2000) and the Potsdam Consultation on Meta-Analysis (Cook et al., 1995). Items from these reporting standards were combined and adapted to make them more appropriate for psychologists (American Psychological Association Publications and Communications Board Working Group on Journal Article Reporting Standards, 2008).

In addition to judging the quality of reporting, reporting standards have other benefits. First, the standards could improve the conducting of reviews, since reviewers are better aware of the required elements of the review process. Second, reviews could be replicated more easily when the research process is clearly reported in the article. Third, the standardization of reporting makes comparison between reviews more convenient (American Psychological Association Publications and Communications Board Working Group on Journal Article Reporting Standards, 2008).

Despite the availability of guidelines for reporting, the extent to which they are used in industrial and organizational psychology research and whether they improved reporting practices remains unknown. However, several studies were conducted to show the state of practice and reporting in systematic reviews and meta-analyses, see studies 6–10 in Table 1.

As found in the studies of Aytug et al. (2012), Kepes et al. (2013), Ahn et al. (2012), Dieckmann et al. (2009), and Fehrmann and Thomas (2011), the reporting quality of different stages in the meta-analytic and/or systematic review process was often unsatisfactory, which could threaten the transparency and replicability of the studies. As becomes clear from the findings on methodological choices in Table 1, a lot of methodological choices are made during the meta-analytic process, which makes it crucial to report transparently about these decisions. This enables other researchers to assess the methodological quality of studies and to replicate the findings, which could result in the accumulation of knowledge. In addition, transparency improves the credibility and generalizability of meta-analytic findings, according to Ahn et al. (2012). The authors recommend the use of reporting guidelines and they encourage researchers to extensively report the stages in the research process. The goal of the current review is to examine the reporting quality of the method section of systematic reviews and meta-analyses in the field of industrial and organizational psychology^1^ since the MARS was launched, i.e., from 2009 to 2016.

Since Aytug et al. (2012) selected some items of the MARS to assess the reporting quality of meta-analyses retrospectively, it is possible to compare reporting quality over time for the same items as Aytug et al. (2012) and to report about additional items that were not evaluated in their study. The same holds for the study Dieckmann et al. (2009), which did not focus particularly on I/O psychology, but still provides results for comparison.

The current study focuses on the quality of reporting in the method section of published articles, which is regarded as the section were the majority of judgment calls are made (Wanous et al., 1989; Dieckmann et al., 2009), such as in the search, selection and coding of systematic reviews and meta-analyses. Most differences between meta-analyses are found in this section (Nieminen et al., 2011), and decisions in this stage, such as for inclusion criteria, seem to be most influential on the final results (Ada et al., 2012). Also the relevance of reporting in the method section, especially in the search, is stressed by several researchers, such as Fehrmann and Thomas (2011).

Yet, the state of reporting in systematic reviews and meta-analyses could be improved according to previous studies. The use of a guideline such as MARS is recommended to increase the consistency and standardization of reporting. Fehrmann and Thomas (2011) showed that the Cochrane guidelines, which are developed in the field of medicine, resulted in better reporting of the computer searches in systematic reviews. A guideline like MARS, developed for the discipline of psychology, is therefore expected to be a useful tool to improve reporting practices in this field.

Because authors of more recent studies might be more familiar with reporting guidelines like MARS, we expected that the overall reporting quality of systematic reviews and meta-analyses improved in the period 2009–2016. That is, we expected that publication year had a positive effect on the overall reporting quality of systematic reviews and meta-analyses in the time period 2009–2016. Furthermore, we expected that systematic reviews and meta-analyses published in journals with a higher journal impact factor (JIF) at the moment of publication had on average a better reporting quality score.

## Methods

### Search strategy

We used a systematic search to identify relevant systematic reviews and meta-analyses in the field of I/O psychology. First of all, we used the Social Sciences Edition of the Journal Citation Reports 2014 (Thomson Reuters, 2016, March 3) to select journals in I/O psychology. The category “Psychology, applied” was selected to search in. We selected the top 10 journals about I/O psychology based on their impact factors. The relevance of the journals was based on their titles and the aims and scope. Finally, we selected the following journals starting with the journal with the highest impact factor: *Journal of Applied Psychology, Personnel Psychology, Journal of Organizational Behavior, Journal of Vocational Behavior, Journal of Occupational Health Psychology, Work and Stress, Organizational Behavior and Human Decision Processes, European Journal of Work and Organizational Psychology, Journal of Business and Psychology and Journal of Occupational and Organizational Psychology*. We also contacted an expert in the field of I/O psychology to verify the inclusion of the most important journals for our search.

We searched these journals electronically to find systematic reviews and meta-analyses between January 2009 and April 2016, using the following keywords, based on Aytug et al. (2012): meta-analysis, meta-analytic, meta^*^, systematic review and system^*^. These publication years were chosen because the study of Aytug et al. (2012) included articles until December 2008. The reporting guidelines PRISMA and MARS were introduced in 2008 and 2009, so we wanted articles after the introduction of these guidelines. More details about the specific searches in each journal can be found in the online Supplementary Material.

### Eligibility criteria and study selection

We defined inclusion/exclusion criteria before conducting the literature search and selection process. First, we included studies about work psychology, industrial psychology, organizational psychology, organizational behavior, vocational psychology and personnel psychology. We excluded studies with content that did not belong to one of the above mentioned fields. Second, we included quantitative systematic reviews and meta-analyses. We excluded quantitative meta-analyses with no literature search for primary studies, for example because they searched for datasets in data archives instead of primary research reports like in the study of Huang et al. (2014). Also quantitative meta-analyses of which the method section could not be assessed with MARS were excluded, for example the study of Lee et al. (2014) where advanced bibliometric mapping techniques were used. We decided to exclude these studies, because they were not representative of the sample of systematic reviews and meta-analyses which we usually see in the field of psychology and to which we wanted to generalize our results. We excluded reviews that were not conducted in a systematic way, narrative reviews, primary studies, reviews of previous systematic reviews or meta-analyses, commentaries or other research designs which were not a systematic review or meta-analysis. Third, we excluded methodological studies, because these were not relevant for our review, such as the study of Aguinis and Gottfredson (2010) about estimating interaction effects. Finally, we excluded articles which were not published in 2009–2016.

Both authors were involved in the study selection process, by screening the titles and abstracts and reading the full-texts of reviews that seemed to be eligible or reviews for which there were still doubts. After the full-text review, a random sample of eligible studies was selected for the systematic review. The Covidence online systematic review platform (Covidence, 2015) was used to store the search results and to support the systematic review process.

### Data extraction

For the data extraction we developed a coding guideline (see Table 4 in the Supplementary Material) with the aim of making the coding decisions more consistent. The first author coded all the retrieved articles with the help of the developed coding guideline. After this first coding occasion, difficulties were discussed and solved by the authors. The first author then improved the coding guideline by adding notes to MARS, where the coding decision was not straightforward based on the item description only, and coded all the articles a second time. After these steps, the second author coded a random sample of articles to check for consistency. Cohen's kappa was used to calculate the coding agreement.

We extracted general information from the retrieved articles: publication year, title, authors, source of the article, if the study was published in an APA journal, type of study (systematic review and/or meta-analysis), topic of the article, number of primary studies included, the use of a reporting guideline and the journal impact factor (JIF) of the year of publication of the articles. In addition, we extracted information with the help of the MARS reporting guideline (American Psychological Association Publications and Communications Board Working Group on Journal Article Reporting Standards, 2008). Our coding guideline consisted of items of the method section of MARS, like inclusion and exclusion criteria, moderator and mediator analyses, search strategies and the coding procedure. After the first coding occasion, several items were adapted or specified to facilitate the coding decision. We used the method section because of the reasons specified in the introduction. Information in this section is the most useful for other researchers who want to replicate the search and selection process of a systematic review. Therefore transparency in the reporting of the search process is a crucial part. Besides this, using the complete MARS guideline was beyond our scope.

We coded for compliance with our coding guideline (see Table 4 in the Supplementary Material) based on MARS by assessing the reporting of the items in the method section of studies. The items could be coded with “Yes,” “No,” “Partial,” or “Unclear.” Items could be coded with “Yes” if the item was fully reported or with “No” when there was no reporting of the item at all. There was also a possibility to code with “Partial” when the item was partially reported, but did not contain the full information stated by the item. Finally, we added the option “Unclear,” for items for which the coding decision was difficult to make. For example, the first item was as follows: “Operational characteristics of independent and dependent variables.” If all main variables were operationalized in a clear and extensive way, the item could be coded as “Yes.” If the operationalization of the variables was not complete, the item could be coded as “Partial.” If there is no reporting of the operationalization of the variables at all, the item was coded as “No.” We noticed that the way in which specific items were reported, differed between studies, and therefore the coding decision was not straightforward in all cases. Because of this, we added notes to each item, with a more elaborate description of possible situations and how to code these. These notes were based on our experience during the coding process and served as operationalizations to clarify the items. By adding these descriptions, we made our coding decisions during this review more transparent. The MARS items should be self-explanatory, but if this is not the case the additional notes could provide clarification. The “Unclear” option was removed on the second coding occasion, by the two coders discussing the “Unclear” items.

### Analysis of the results

The objective of this systematic review was to examine the reporting quality of the method section of systematic reviews and meta-analyses in 2009–2016 in the field of I/O psychology and to test our hypotheses. We used descriptive statistics to present the reporting quality of systematic reviews and meta-analyses by showing compliance with the items in the coding guideline based on MARS (Table 4 in the Supplementary Material). We presented general information about the retrieved systematic reviews and meta-analyses. We calculated total scores for all studies in our review to measure the overall reporting quality. The values “Yes,” “Partial,” and “No,” were replaced by numeric values. So the values became “Yes” = 1, “Partial” = 0.5, and “No” = 0. The maximum possible score was 22. Because some MARS items are more relevant and elaborate than others, the reporting percentages of the different items are more valuable than the overall reporting quality scores.

In addition, a cross-classified multilevel regression analysis was conducted, because of the multilevel structure of the data. The sample of 120 systematic reviews and meta-analyses was nested within 93 different authors; some first authors had multiple studies within the sample. Besides that, there existed a multilevel structure of journals, because all 120 studies came from the 10 selected journals. By analyzing the data with a cross-classified model, we could account for the two multilevel structures in our data. This could not be done in a regular multilevel model, because authors and journals were not hierarchically ordered. At the article level, we tested whether publication year and JIF were positively associated with the overall reporting quality scores.

## Results

### Literature search and study selection process

**Figure 1:**
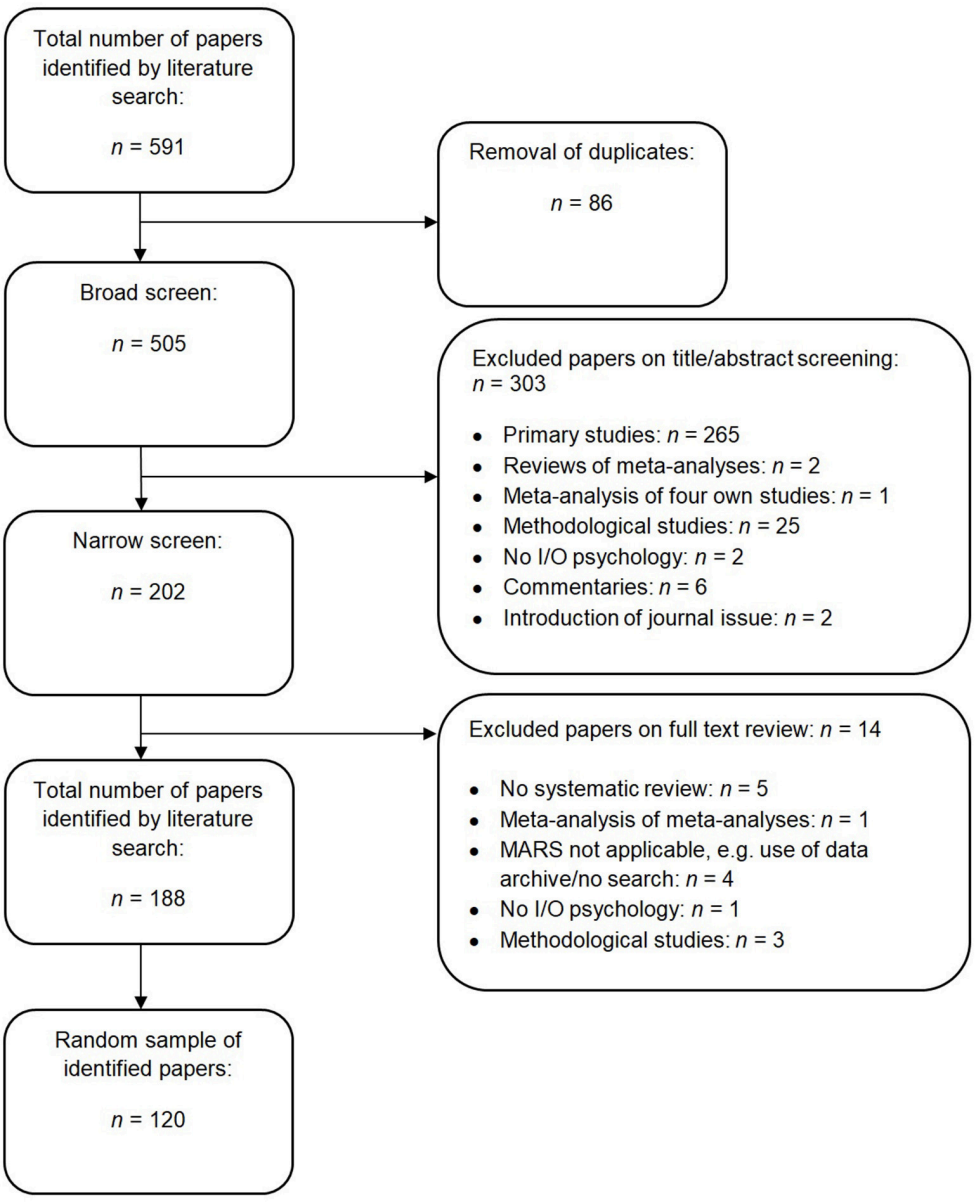
Flow chart of the literature search and selection process of the review.

The flow chart in Figure 1 depicts the literature search and selection process in the review. A total of 591 systematic reviews and meta-analyses were retrieved, of which 86 papers were excluded as duplicates. Of the 505 papers left, all the titles and abstracts were screened by the first author, and a random sample of 60 papers (12%) by the second author; 303 were excluded based on the inclusion/exclusion criteria. In the full-text screening of 202 papers, 14 were excluded based on the criteria. For the specific exclusion reasons in both the broad and narrow screening, see Figure 1. Thus 188 papers were identified by the literature search. We selected a random sample of 120 papers for our review.

### Coding agreement

We used Cohen's kappa to calculate the agreement between the two coding occasions for the first author and to calculate the inter-coder agreement between the two authors. The agreement between the two coding occasions of the first author was κ = 0.91. The majority of differences between the two occasions were due to adaptations of the coding guideline. The second coder coded a random sample of 25 articles (21% of the sample) to check for consistency. The inter-coder agreement was κ = 0.97. The few discrepancies between the coders were solved through discussion.

### Characteristics of included studies in the review

We analyzed a random sample of 120 systematic reviews and meta-analyses from the field of I/O psychology to assess the reporting quality of the method section with MARS. The sample characteristics are presented in Table 2. Most reviews were published in the Journal of Applied Psychology (43), the journal with the highest impact factor of the top 10 journals. In addition, the majority of studies were meta-analyses, with or without a systematic review (117), except for the systematic reviews of Deligkaris et al. (2014), Leitão and Greiner (2016), and Robertson et al. (2015). Reporting standards like PRISMA or MARS were almost never used in the 120 retrieved studies. With exception of the systematic review of Deligkaris et al. (2014) that used the PRISMA statement in their research and the meta-analyses of Salgado and Táuriz (2014) and Stajkovic et al. (2015) that mentioned MARS, and used it for an extensive description of the included primary studies. Because only these three studies reported about the use of reporting standards, it was not possible to do any additional analysis for the use of reporting standards.

**Table 2:** Characteristics of systematic reviews and meta-analyses in the review (*n* = 120).

**Characteristics**	***n (%)***
**SOURCE**	
Journal of Applied Psychology	43 (35.8)
Personnel Psychology	11 (9.2)
Journal of Organizational Behavior	12 (10.0)
Journal of Vocational Behavior	19 (15.8)
Journal of Occupational Health Psychology	5 (4.2)
Work and Stress	7 (5.8)
Organizational Behavior and Human Decision Processes	5 (4.2)
European Journal of Work and Organizational Psychology	5 (4.2)
Journal of Business and Psychology	6 (5.0)
Journal of Occupational and Organizational Psychology	7 (5.8)
**APA**	
Yes	48 (40.0)
No	72 (60.0)
**PUBLICATION YEAR**	
2009	14 (11.7)
2010	16 (13.3)
2011	22 (18.3)
2012	14 (11.7)
2013	16 (13.3)
2014	13 (10.8)
2015	18 (15.0)
2016^a^	7 (5.8)
**RESEARCH DESIGN**	
Systematic review	3 (2.5)
Meta-analysis (with and without a systematic review)	117 (97.5)

a *The literature search was conducted through April 2016*.

### Reporting quality of systematic reviews and meta-analyses based on MARS

For the purpose of assessing the reporting quality of systematic reviews and meta-analyses in the field of I/O psychology, we coded the retrieved studies with our coding guideline (Table 4 in the Supplementary Material) based on MARS. The compliance with MARS is presented in Table 3, by showing the percentages of “Yes,” “Partial” and “No” for each item and will be described below. The data from the coding process are available in the online Supplementary Material.

**Table 3:** Compliance with the coding guideline for reporting quality based on MARS (American Psychological Association Publications and Communications Board Working Group on Journal Article Reporting Standards, 2008) (*n* = 120).

**Item**	**Section**	**Characteristic**	***n (%)***		
			**Yes**	**Partial**	**No**
**METHOD**					
1	Inclusion and exclusion criteria	Operational characteristics of independent (predictor) and dependent (outcome) variable(s)	50 (41.7)	32 (26.7)	38 (31.7)
2		Eligible participant populations	35 (29.2)		85 (70.8)
3		Eligible research design features (e.g., random assignment only, minimal sample size)	35 (29.2)		85 (70.8)
4		Time period in which studies needed to be conducted/published	7 (5.8)	1 (0.8)	112 (93.3)
5		Geographical and/or cultural restrictions	2 (1.7)		118 (98.3)
6	Moderator and mediator analyses	Definition of all coding categories used to test moderators or mediators of the relation(s) of interest	52 (43.3)	18 (15.0)	50 (41.7)
7	Search strategies	Reference and citation databases searched	117 (97.5)		3 (2.5)
8		Keywords used to enter databases and registries	105 (87.5)	4 (3.3)	11 (9.2)
9		Time period covered by the search	28 (23.3)	55 (45.8)	37 (30.8)
		Other efforts to retrieve all available studies:			
10		• Listservs queried	29 (24.2)		91 (75.8)
11		• Contacts made with authors	47 (39.2)		73 (60.8)
12		• Reference lists of reports examined	80 (66.7)		40 (33.3)
13		Method of addressing reports in languages other than English	21 (17.5)		99 (82.5)
		Process for determining study eligibility:			
14		• Aspects of reports were examined (i.e., title, abstract, and/or full text)	16 (13.3)	2 (1.7)	102 (85.0)
15		• Number and qualifications of relevance judges	1 (0.8)	7 (5.8)	112 (93.3)
16		• Indication of agreement, how disagreements were resolved	1 (0.8)	3 (2.5)	116 (96.7)
17		Treatment of unpublished studies	74 (61.7)		46 (38.3)
18	Coding procedures	Number and qualifications of coders (e.g., level of expertise in the area, training)	12 (10.0)	72 (60.0)	36 (30.0)
19		Inter-coder reliability or agreement	80 (66.7)		40 (33.3)
20		Whether each report was coded by more than one coder and if so, how disagreements were resolved	80 (66.7)	2 (1.7)	38 (31.7)
21		Assessment of study quality	2 (1.7)		118 (98.3)
22		How missing data were handled	109 (90.8)		11 (9.2)

We coded the reporting quality of the articles with our coding guideline with 22 items (see Table 4 in Supplementary Material) about the method section. The guideline was based on MARS, but we adapted some items and added notes to make it more user-friendly, as described in Section Data Extraction. The method section consisted of four main sections in the coding guideline, which will be described below:

- Inclusion and exclusion criteria: The operational characteristics of independent and dependent variables (item 1) were fully reported in 42% of the studies. Studies were coded as “Partial” when only a part of the variables were operationalized or when the operationalizations of the variables were limited, which means that the definition of the variables is reported in such a way, that it is not clear to the reader; 27% of the studies were coded as “Partial.” The eligible participant populations (item 2) and eligible research design features (item 3) were both fully reported in 29% of the studies. In addition, the time period in which primary studies needed to be conducted/published (item 4) was reported for 6% of the reviews, and the geographical and/or cultural restrictions (item 5) in only 2% of the studies.
- Moderator and mediator analyses: The definitions of all coding categories used to test moderators or mediators (item 6) were fully reported in 43% of all reviews. Studies were coded with “Partial” when this description was very short and therefore not clear for the reader or when only a part of the moderators/mediators was defined; this was the case in 15% of the studies.
- Search strategies: 98% of the review authors reported the reference and citation databases searched (item 7) and 88% the keywords used in databases and registries (item 8). The time period that covered the search (item 9) was fully reported in 23% of the studies. The time period was coded as “Partial” when merely the date of the search was reported or only the starting date of the time period in which the search was conducted; 46% of the reviews were coded with “Partial.” Efforts to retrieve all available primary studies were reported in 24% of the reviews for listservs (item 10), in 39% for contacts made with authors (item 11), and in 67% for reference lists of reports examined (item 12). Methods of addressing reports in languages other than English (item 13) were reported in 18% of the reviews. In addition, the aspects of reports that were examined (item 14) for determining study eligibility were only reported in 13% of the reviews. The number and qualifications of relevant judges (item 15) and an indication of agreement (item 16) were almost never reported (both 1%). The treatment of unpublished studies (item 17) was reported in 62% of the reviews.
- Coding procedures: The number and qualifications of coders (item 18) were fully reported in 10% of the reviews. Most studies that were coded as “Partial” only reported the number of coders, but not their qualifications; this was the case in 60% of the reviews. The inter-coder reliability (item 19) and resolving of disagreements (item 20) were both coded for 67% of the reviews. However, the assessment of study quality (item 21) was reported in 2% of the reviews. Finally, the treatment of missing data (item 22) was reported in 91% of the reviews.

### Overall reporting quality, publication year and journal impact factor

For the 120 systematic reviews and meta-analyses, we calculated a total score for reporting quality based on the coding guideline. The highest possible score was 22, but we found no study with such a score. The mean score of the 120 studies was 9.0. The minimum and maximum scores in our review were 3.0 and 15.0. The scores for reporting quality were normally distributed in our sample.

We did a cross-classified multilevel analysis to test whether publication year and JIF significantly predicted the reporting quality scores of articles. The results showed that a cross-classified multilevel analysis was necessary, because a substantial amount of the variance existed at the author and journal level. The intra-class correlation (ICC) was ρ = 0.168 for authors and ρ = 0.120 for journals, which is the total proportion of variance in reporting quality scores at the author level and the journal level. Together both levels account for 0.288 of the total variance. With a significance level of α = 0.05, publication year was not a significant predictor of the reporting quality scores of the articles, with *b* = 0.062, *t*_(17)_ = 0.671, *p* = 0.511. The effect of JIF on the reporting quality scores of articles was also not significantly different from 0, with *b* = 0.456, *t*_(17)_ = 1.786, *p* = 0.092. Therefore both hypotheses could be rejected.

## Discussion

The goal of this systematic review was to examine the reporting quality of the method section of systematic reviews and meta-analyses from 2009 to 2016 in the field of I/O psychology, and in this way to update previous reviews, such as those of Aytug et al. (2012) and Dieckmann et al. (2009). Because Aytug et al. (2012) assessed meta-analyses published between 1995 and 2008 and Dieckmann et al. (2009) between 1994 and 2004, when PRISMA or MARS did not yet exist, we expected that the overall reporting quality would have been improved since then. We also expected that publication year had a positive effect on the overall reporting quality during the time period 2009–2016. In addition, we expected that journals with a higher JIF had on average a higher reporting quality score.

We assessed the method section of a random sample of 120 systematic reviews and meta-analyses published in 10 different journals of I/O psychology, with the help of MARS. We chose this section because information about the methodology is the most useful for a researcher who wants to replicate the search and selection process and because different researchers stressed the importance of clear reporting in this section (Wanous et al., 1989; Dieckmann et al., 2009; Fehrmann and Thomas, 2011; Nieminen et al., 2011; Ada et al., 2012).

Despite the introduction of several reporting guidelines, the results showed that the overall reporting quality in the method section of systematic reviews and meta-analyses in the sample was on average low (9 out of 22). There still existed a lot of variation in the reporting of the 22 assessed items, see Table 3. In the next sections we will discuss the strong and weak areas of reporting in different parts of the method section in systematic reviews and meta-analyses in I/O psychology, and will reflect on our expectations. Finally, we will discuss some limitations and possibilities for future research.

### Inclusion and exclusion criteria

In general the current results concerning this section of MARS are in line with Aytug et al. (2012): many studies did not provide full information about these issues. The operational characteristics of independent and dependent variables (item 1) were not reported in almost one-third of the studies. One possible reason might be that review authors had previously operationalized these variables in the introduction. This could be checked in future research. However, according to the guideline MARS, this should actually be reported in the method section. Therefore we can still conclude the reporting was insufficient. Studies often reported extensively about inclusion/exclusion criteria, which is in line with the result of Dieckmann et al. (2009) on this item. Other items considered relevant in MARS were not frequently reported in our sample, like eligible participant populations (item 2), eligible research design features (item 3), and the time period in which primary studies needed to be conducted or published (item 4). In addition, the reporting of geographical or cultural restrictions (item 5) was low (1.7%), but this item might be less relevant to I/O psychology with respect to the content of articles in that field. Missing information in this section of a report is problematic, since this information is required for replicating the review.

### Moderator and mediator analyses

In line with the findings by Ahn et al. (2012) we found that the formulation of operational definitions of moderators (item 6) was only reported in 43.3% of the articles. However, for this item it might be the case that moderators or mediators were described in the introduction or that these studies did not conduct a moderator/mediator analyses at all. Dieckmann et al. (2009) found that almost two-thirds of the meta-analyses in their review reported a complete coding scheme for the moderators in the study, and most of the other meta-analyses described some aspects of it. In order to make studies more informative and replicable, the definition of all coding categories used to test moderators or mediators should be improved, which is also stated by Dieckmann et al. (2009).

### Search strategies

The reporting of the used keywords in searched databases (item 7) improved in comparison with Aytug et al. (2012); they found 40% for this item in comparison with 88% in our review. In the study of Fehrmann and Thomas (2011) the majority of systematic reviews reported the use of alternate search terms, which are part of the final keywords used in the search. The time period covered by the search (item 9) was merely reported in 23.3% of the articles. The date of the search or the starting date of the time period in was searched was often reported, but full information about the time period that was covered by the search was often lacking. This is precisely the information that is needed for replication of the search. In the study of Aytug et al. (2012) the time period that was covered by the search was only reported in 12% of the studies, so there is some improvement in our review.

Efforts to retrieve all available studies, like listservs (item 10) used, contacts made with authors (item 11) or reference lists of reports examined (item 12), were reported in some reviews. However, the evaluation of the reporting quality of these items was not easy, because we were not able to judge if these efforts were actually made. The treatment of reports in languages other than English (item 13) was merely reported in 17.5% of the studies, the reasons for this are yet unclear, but this item is crucial for replication of the search.

The process to judge study eligibility was reported very poorly: only 13.3% of the reviews reported which aspects of reports were examined (item 14), and 0.8% of the reviews fully reported the number and qualifications of the judges (item 15) or the indication of agreement and the resolving of disagreements (item 16). This poor reporting threatens the transparency and replicability of the research. With this information lacking, it is not possible to evaluate the risk of bias due to study selection.

A reasonable amount of systematic reviews and meta-analyses reported the inclusion of unpublished studies and what efforts were made to get access to them (item 17). However, not all reviews reported about the efforts made to obtain unpublished studies. In the study of Dieckmann et al. (2009) half of the meta-analyses reported about the inclusion of unpublished studies, but no conclusions can be drawn about the treatment of these unpublished studies. It is important to report both items in order to assess the risk of publication bias, and to enable researchers to replicate the search and selection process.

### Coding procedures

The number and qualifications of the coders (item 18) was fully reported in 10% of the reviews, and partially for 60% of the reviews. The majority probably reported the number of coders and not their qualifications, which was comparable to the result found by Aytug et al. (2012) that 57% of the reviews reported the number of coders. The study of Dieckmann et al. (2009) found that 43% reported the number of coders. Only two-thirds of the reviews reported the inter-coder agreement (item 19), and reported if the included primary studies were coded by more than one coder and if so, how disagreements were solved (item 20). In Dieckmann et al. (2009) only 34% of the meta-analyses reported a measure of coding reliability. By reporting above mentioned items, the risk of bias due to the coding could be assessed. In line with Aytug et al. (2012) and Kepes et al. (2013) we found that the assessment of study quality (item 21) was merely reported in two systematic reviews, which is very concerning. We question whether reviewers conduct quality assessments of included studies at all. Related to this, is the finding of Dieckmann et al. (2009) that half of the meta-analyses reported insufficient reporting in the primary studies they included. Finally, the majority of the systematic reviews and meta-analyses reported about the treatment of missing data (item 22), which improves the replicability of the research.

### Publication year and journal impact factor

We failed to support the first hypothesis that more recent systematic reviews and meta-analyses were more familiar with reporting guidelines like MARS or PRISMA, and therefore had a higher overall reporting quality. We found no significant effect of publication year on the reporting quality scores of articles. A possible reason why a significant increase in the time period 2009–2016 is lacking, could be that researchers are still not aware of the existence of guidelines such as MARS or PRISMA, because in our sample only three reviews mentioned to use MARS or PRISMA. In addition, a quick and dirty search in Google Scholar for the original MARS article from American Psychological Association Publications and Communications Board Working Group on Journal Article Reporting Standards (2008) resulted in only 43 citations in total, which is an indication that it is not well-known. In contrast to our result, Dieckmann et al. (2009) found that recent meta-analyses in the sample from 1994 to 2004 in their review reported more thorough searches of the literature.

Aytug et al. (2012) found that meta-analyses with better SSCI rankings had a better reporting quality than journals with less preferable SSCI rankings. SSCI rankings could be considered as a measure of journal quality, like the journal impact factor. Dieckmann et al. (2009) tested the relationship between JIF and several reporting and practice variables, and found no significant result, although with the use of a percentage adherence score for the meta-analyses, they did find a moderate relationship with JIF. Their explanation for the result was that journals with higher JIFs require their researchers more to use reporting and practice guidelines. However, we failed to support our second hypothesis that JIF was a positive predictor of the reporting quality scores of articles.

### The reporting of processes and findings

In addition to the above findings obtained by applying the MARS, we noticed that although most reports had separate method and results sections, many studies reported about the study selection process and the outcome of this selection process in the method section simultaneously. This makes it impossible to distinguish between inclusion and exclusion criteria that were formulated in advance, and final inclusion and exclusion decisions that were made during the data collection. Therefore we recommend researchers to separate processes and findings, and adhere to the usual arrangement of method and results sections.

### Study limitations and future research

Since the replicability of the systematic reviews and meta-analyses based on their reports was of our main interest in the current study, we focused on the method sections of the evaluated studies. This gives an indication of the reporting quality, although this is not optimal because there could be variation in reporting between the different sections of the research report. It would be interesting to examine compliance with the complete MARS in the future to get a clear overview of the reporting quality on all the sections in the research report.

### Limitations of reporting guidelines

The use of reporting guidelines has some limitations. For example, MARS consists of crucial items to report in a research report, however the exact definition of some items remains unclear. We solved this problem by adding notes to the items in our coding guideline (Table 4 in the Supplementary Material), but there still exists the risk that other researchers will interpret the items in a different manner. Besides this, the reporting quality could be assessed with MARS, but not the methodological quality. So articles with low reporting scores, can still have a high methodological quality. In addition, some MARS items are more relevant to report than others, therefore the reporting percentages of different items are more valuable than the overall reporting quality scores we calculated. We consider items in the categories of inclusion and exclusion criteria and the search strategy as most crucial to report, because reporting these enables replication by other researchers. However, items in the category of coding procedures are important as well to assess the methodological quality of studies. Yet, the development of a weighting scheme for the items would be valuable, for example creating a composite score for subitems such as 10, 11, and 12 would be more appropriate. Besides that, reporting the complete MARS could fill articles with irrelevant information and too many details (American Psychological Association Publications and Communications Board Working Group on Journal Article Reporting Standards, 2008). The standards might also lead to a loss of uniqueness. This means that authors just report according to the guidelines, and leave out interesting findings which are unique and specific for their study. The research methodology can vary between studies, and therefore the information that is relevant to report also differs as well (American Psychological Association Publications and Communications Board Working Group on Journal Article Reporting Standards, 2008).

We recommend researchers to use complete reporting guidelines, primarily to check off all the items in the guidelines for their research, and to report items which they considered as applicable and crucial for their studies. Additional efforts should be made to implement the available guidelines in research practice by publishing instructional articles to promote and clarify the use of the guidelines.

## Conclusion

In summary, the goal of this systematic review was to examine the reporting quality in the method section of systematic reviews and meta-analyses from 2009 to 2016 in the field of I/O psychology with the help of APA's MARS. The results indicate that the overall reporting quality is still unsatisfactory, and that reporting of items in the method section of reviews varies a lot. We found no effect of publication year and JIF on the reporting quality scores of articles. We compared our results on the items in the method section with the items reported in Aytug et al. (2012) and Dieckmann et al. (2009). Like Aytug et al. (2012) we found that the reporting quality of the inclusion and exclusion criteria was not sufficient, and found no improvement. The reporting of moderator and mediator analyses still needs improvement as well, which was already stated by the previous studies of Ahn et al. (2012) and Dieckmann et al. (2009). However, we found some improvements in items in the search strategy in comparison with Aytug et al. (2012). For both the study of Aytug et al. (2012) as the study of Dieckmann et al. (2009) improvements were found in the coding procedures.

It is crucial to clearly report about processes and findings in research. This will improve the transparency and replicability of the research, and it gives the possibility to assess the methodological quality of studies. Therefore, we recommend researchers to use reporting guidelines for their systematic reviews and meta-analyses, primarily to check for all the items in the guidelines and report the ones which were considered applicable and crucial.

We recommend journals to require their researchers to use MARS or another reporting guideline for their manuscripts, this will improve the reporting quality. Finally, we would like to show the importance of clear reporting and the value of reporting guidelines to researchers, editors, practitioners and students with our review.

## Author contributions

CR developed the initial research plan, which was further developed by both authors. NS did the literature search, the study selection process, the data extraction, the assessment of the reporting quality of the articles, the analyses, the interpretation of the results, the discussion and conclusion. CR contributed in this process by working partially on the study selection process, the data extraction, the assessment of the reporting quality and introduced interesting discussion points. Furthermore, CR contributed to the writing of the article. During the research process, the authors simultaneously discussed and evaluated the work. Both authors give final approval of the version to be published. The authors agree to be accountable for all aspects of the work in ensuring that questions related to the accuracy or integrity of any part of the work are appropriately investigated and resolved.

### Conflict of interest statement

The authors declare that the research was conducted in the absence of any commercial or financial relationships that could be construed as a potential conflict of interest.

## Footnotes

^1^With the term “industrial and organizational psychology” we also refer to the related fields of work psychology, organizational behavior, vocational psychology and personnel psychology. In this systematic review we will abbreviate the term to “I/O psychology.”
