# Supplementary material for: The Reporting Quality of Systematic Reviews and Meta-Analyses in Industrial and Organizational Psychology: A Systematic Review
Source: Front Psychol. 2017 Aug 22;8:1395. doi: 10.3389/fpsyg.2017.01395 (PMC5572251; doi:10.3389/fpsyg.2017.01395)
Supplement: Supplementary file 1 [file Table4.docx]

Supplementary Material

**The Reporting Quality of Systematic Reviews and Meta-Analyses in Industrial and Organizational Psychology: A Systematic Review**

Naomi Schalken*, Charlotte Rietbergen

*** Correspondence:** naomischalken@gmail.com

**Supplementary Material 1 – Coding guideline**

The coding guideline in Table 4 should be used as follows:

Each item has only one answer option, which could be ‘Yes’, ‘Partial’, ‘No’ or ‘Unclear’. Items can be coded with ‘Yes’ if the item is fully reported or with ‘No’ when there is no reporting of the item at all. The ‘Partial’ option can be used if the item is partially reported, or with ‘Unclear’ for items for which the coding decision is difficult to make. For example, the first item is as follows: ‘Operational characteristics of independent and dependent variables’. If all main variables are operationalized in a clear and extensive way, the item can be coded as ‘Yes’. If the operationalization of the variables is not complete, the item can be coded as ‘Partial’. If there is no reporting of the operationalization of the variables at all, code the item as ‘No’. In case of difficult coding decisions, the additional notes could provide clarification about the items.

Table 4

*Coding guideline for the reporting quality based on MARS (APA, 2008)*

| Item | Section | Characteristic | Yes | No | Partial | Unclear |
| --- | --- | --- | --- | --- | --- | --- |
|  | Method |  |  |  |  |  |
| 1 | Inclusion and exclusion  criteria | Operational characteristics of independent (predictor) and dependent (outcome) variable(s)  *Yes: when the measurement/definition of the variables is clearly reported. The variables could be extracted from the research question(s) and hypotheses in the study.*  *Yes: when an extensive description of the categorization/coding of the variables in the study is reported.*  *Yes: when there is explicitly referred to operationalizations in another section in the article, for example the introduction or a table.*  *Partial: when there is a limited/very short description of the coding/categorization/definition of the variables in the study, which means that the coding/categorization/definition of the variables is not directly clear to the reader.*  *Partial: when there is a description of the coding/categorization/definition of a part of the variables in the study.* | … | … | … | … |
| 2 |  | Eligible participant populations  *Yes: when there is reported that there were no restrictions for populations.* |  |  |  |  |
| 3 |  | Eligible research design features (e.g., random assignment only, minimal sample size)  *Yes: when eligible research designs are described, for example experimental, cross-sectional or longitudinal designs were included/excluded.*  *Yes: when research design features are described, for example only studies with a control group were eligible.*  *Yes: when there is reported that there was no restriction for study design.* |  |  |  |  |
| 4 |  | Time period in which studies needed to be conducted/published.  *Yes: when there is reported that there was no time restriction for studies.*  *Partial: when not the whole time period is reported, but only one year.* |  |  |  |  |
| 5 |  | Geographical and/or cultural restrictions  *Yes: when there is reported that there were no geographical/cultural restrictions.* |  |  |  |  |
| 6 | Moderator and mediator analyses | Definition of all coding categories used to test moderators or mediators of the relation(s) of interest  *Yes: when an extensive description of the coding/categorization/definition of the moderators/mediators is reported in the study.*  *Partial: when a limited/very short description of the coding/categorization/definition of the moderators/mediators is reported in the study, which means that the coding/categorization/definition of the moderators is not directly clear to the reader.*  *Partial: when an extensive description of the coding/categorization/definition of a part of the moderators/mediators is reported.* |  |  |  |  |
| 7 | Search strategies | Reference and citation databases searched  *No: when there is only described that they used databases, but not which databases.* |  |  |  |  |
| 8 |  | Keywords used to enter databases and registries  *Yes: when there is referred to the keywords in a table or another section in the article.*  *Partial: when examples of keywords are reported or a part of the keywords.* |  |  |  |  |
| 9 |  | Time period covered by the search  *Yes: when the time period in which studies are electronically searched is reported.*  *Yes: when there is reported that they used the entries of the databases till a specific date for the search.*  *Partial: when the time period of a part of the search is reported, for example for the manual search.*  *Partial: when the date of the search is reported.*  *Partial: when the date/year is reported from which the search is conducted, for example ‘studies after 1985’.*  *No: when there is reported that there was no specific time period in which studies were searched.* |  |  |  |  |
| 10 |  | Other efforts to retrieve all available studies:   - Listservs queried *(electronic mailing lists or discussion lists)* |  |  |  |  |
| 11  12 |  | - Contacts made with authors   *Yes: When there is reported that contact was made with authors to retrieve studies, for example unpublished studies.*  *No: When there is reported that contact was made with authors to retrieve additional data/information about studies.*  *No: When there is reported that contact was made with authors via a listserv, then ‘yes’ for item 10a.*   - Reference lists of reports examined |  |  |  |  |
| 13 |  | Method of addressing reports in languages other than English  *Yes: when there is reported that reports in languages other than English were included/excluded.* |  |  |  |  |
| 14 |  | Process for determining study eligibility:   - Aspects of reports were examined (i.e, title, abstract, and/or full text)   *Yes: when the description of which aspects were examined is reported in the search and/or in the inclusion and exclusion criteria.*  *Partial: when the examined aspects of reports are only described for a part of the search, for example only in the manual search.* |  |  |  |  |
| 15 |  | - Number and qualifications of relevance judges   *Partial: when only the number of judges is reported, and not their qualifications.* |  |  |  |  |
| 16 |  | - Indication of agreement, how disagreements were resolved   *Partial: when there is only reported about how disagreements were resolved, and not about the indication of agreement (or the other way round).* |  |  |  |  |
| 17 |  | Treatment of unpublished studies  *Yes: when there is explicitly reported about the search/treatment of unpublished studies and how this was done.*  *Yes: when there is reported about the exclusion of unpublished articles or inclusion of only published articles.*  *Yes: when there is reported about the method to prevent publication bias.*  *No: when there is only reported about the inclusion of unpublished studies, but not how they were retrieved.*  *No: when there is only reported about the analysis of publication bias.* |  |  |  |  |
| 18 | Coding procedures | Number and qualifications of coders (e.g., level of expertise in the area, training)  *Yes: when the number of coders and the qualifications (for example training) of all coders are reported, inclusive for the authors.*  *Partial: when the number of coders is reported, but not their qualifications.*  *Partial: when the number of coders is reported, but only a part of the qualifications of the coders.* |  |  |  |  |
| 19 |  | Inter-coder reliability or agreement  *Yes: when there is reported about agreement between coding occasions, in the case of one coder.* |  |  |  |  |
| 20 |  | Whether each report was coded by more than one coder and if so, how disagreements were resolved  *Yes: when there was only one coder, and the discrepancies between two coding occasions were reported.*  *Yes: when there were more coders, but there was reported that they had no disagreement.*  *Partial: when there is reported about the coding of reports by more than one coder, but not how disagreements were resolved (or the other way round).* |  |  |  |  |
| 21 |  | Assessment of study quality  *Yes: when there is reported about quality assessment of primary studies and in which way this was done.* |  |  |  |  |
| 22 |  | How missing data were handled  *Yes: when studies report about the coding of particular articles with missing data.*  *Yes: when studies replace the missing data with particular calculations, for example in the case of missing reliability measures.*  *Yes: when studies excluded articles with missing data, for example when correlation coefficients were not reported.*  *Yes: when authors were contacted when there was missing data.* |  |  |  |  |
