# Supplementary material for: The Reporting Quality of Systematic Reviews and Meta-Analyses in Industrial and Organizational Psychology: A Systematic Review
Source: Front Psychol. 2017 Aug 22;8:1395. doi: 10.3389/fpsyg.2017.01395 (PMC5572251; doi:10.3389/fpsyg.2017.01395)
Supplement: Supplementary file 3 [file DataSheet2.DOCX]

***Supplementary Material 2***

**The Reporting Quality of Systematic Reviews and Meta-Analyses in Industrial and Organizational Psychology: A Systematic Review**

**Naomi Schalken*, Charlotte Rietbergen**

*** Correspondence:** naomischalken@gmail.com

In this document additional information is given about:

1. The systematic search strategy in specific journals
2. Output of the cross-classified multilevel analyses in HLM
3. Calculation of the ICC
4. **Systematic search strategy in specific journals**

In the section we shortly add some specific information about the search in the selected journals. We selected the top 10 journals about I/O psychology based on the impact factors of the journals in the Social Sciences Edition of the Journal Citation Reports 2014 (Thomson Reuters, 2016, March 3).

Search strategy of the included journals:

1. **Journal of applied psychology:** Articles of this journal can be accessed via the search engine Ovid. Details about Ovid can be found below.
2. **Personnel psychology:** Articles of this journal can be accessed via the search engine Wiley online library. Details about the Wiley online library can be found below.
3. **Journal of organizational behavior:** Articles of this journal can be accessed via the search engine Wiley online library. Details about the Wiley online library can be found below.
4. **Journal of vocational behavior:** Articles of this journal can be accessed via the search engine Sciencedirect. Details about Sciencedirect can be found below.
5. **Journal of occupational health psychology**: Articles of this journal can be accessed via the search engine Ovid. Details about Ovid can be found below.
6. **Work & Stress:** Articles of this journal can be accessed via the search engine EBSCOhost. Details about EBSCOhost can be found below.
7. **Organizational behaviour and human decision processes:** Articles of this journal can be accessed via the search engine Sciencedirect. Details about Sciencedirect can be found below.
8. **European journal of work and organizational psychology:** Articles of this journal can be accessed via the search engine Taylor & Francis Online. Details about Taylor & Francis Online can be found below.
9. **Journal of business and psychology:** Articles of this journal can be accessed via the search engine SpringerLink. However, this online library searched in the full texts of articles, which results in search results which are not relevant. Therefore we decided to search in the Journal of business and psychology via Web of Science. The search strategy for Web of Science can be found below.
10. **Journal of occupational and organizational psychology:** Articles of this journal can be accessed via the search engine EBSCOhost. Details about EBSCOhost can be found below.

**Search strategy in the online libraries of:**

- **Ovid:** Go to the Multi-Field Search option. Fill in: Journal of applied psychology (Journal name) AND systematic review OR meta-analysis OR system* OR meta* OR meta-analytic (Abstract). Limits: Publication year: 2009-2016. Do this in the same way for the Journal of occupational health psychology.
- **Wiley online library:** Go to the advanced search option. Fill in Journal of organizational behaviour (Publication Titles) AND systematic review OR meta-analysis OR system* OR meta* OR meta-analytic (Abstract). Specify date range: 2009-2016.
- **Sciencedirect:** Go to the advanced search option. In this journal we searched each time with two search terms (more search terms not possible). The first one was Journal of vocational behaviour (Source title) AND (each time one of the following search terms): systematic review OR meta-analysis OR system* OR meta* OR meta-analytic (Abstract). 2009-2016. The search was conducted in the same way for Organizational behaviour and human decision processes.
- **EBSCOhost:** Go to the search option. Fill in: Work & Stress (Journal name) AND systematic review OR meta-analysis OR system* OR meta* OR meta-analytic (Abstract). Limits: Published data January 2009 –April 2016. Same approach for the Journal of occupational and organizational psychology.
- **Taylor & Francis Online:** Go to the advanced search. Then fill in: European journal of work and organizational psychology (Publication title) AND systematic review OR meta-analysis OR system* OR meta* OR meta-analytic (Abstract). Years: 2009-2016.
- **SpringerLink:** Search could not be easily conducted in this search engine. Therefore Web of Science was used.
- **Web of Science:** Fill in ‘Journal of Business and Psychology’ (Publication Name) AND ‘Systematic review OR system* OR meta-analysis OR meta-analytic OR meta*’. Select 2009-2016.

The search was conducted at 20 April and the Web of Science search at 23 April 2016.

1. **Output of the cross-classified multilevel analyses in HLM**

**Intercept only model**

The maximum number of level-1 units = 120
The maximum number of row-level units = 93
The maximum number of column-level units = 10
The maximum number of iterations = 100

Method of estimation: full maximum likelihood
The maximum number of iterations = 100
Z-structure: independent

The outcome variable is REPORTIN 

### Summary of the model specified

#### Level-1 Model

*REPORTIN_ijk_* = *π_0jk_* + *e_ijk_*

#### Level-2 Model

*π_0jk_* = θ*_0_* + *b_00j_* + *c_00k_*

#### Mixed Model

*REPORTIN_ijk_* = θ*_0_*+ *b_00j_* + *c_00k_* + *e_ijk_*

For starting values, data from 120 level-1, 93 row-level
and 10 column-level records were used

## Final Results - iteration 26

**Iterations stopped due to small change in likelihood function**


σ^2^ = 3.32263

τ_rows_

| INTRCPT1 |
| --- |
| ICPTROW,*b_00j_* |
| 0.78566 |

τ_columns_

| INTRCPT1 |
| --- |
| ICPTCOL,*c_00k_* |
| 0.55760 |

The value of the log-likelihood function at iteration 26 = -2.585232E+002

#### Final estimation of fixed effects:

| Fixed Effect | Coefficient | Standard error | *t*-ratio | Approx. *d.f.* | *p*-value |
| --- | --- | --- | --- | --- | --- |
| For INTRCPT1, *π_0_* | | | | | |
| INTERCEPT,θ*_0_* | 9.052184 | 0.327421 | 27.647 | 17 | <0.001 |

#### Final estimation of row and level-1 variance components:

| Random Effect | Standard  Deviation | Variance  Component | *d.f.* | χ^2^ | *p*-value |
| --- | --- | --- | --- | --- | --- |
| INTRCPT1/ ICPTROW,*b_00j_* | 0.88638 | 0.78566 | 92 | 107.72693 | 0.126 |
| level-1, *e* | 1.82281 | 3.32263 |  |  |  |

#### Final estimation of column level variance components:

| Random Effect | Standard  Deviation | Variance  Component | *d.f.* | χ^2^ | *p*-value |
| --- | --- | --- | --- | --- | --- |
| INTRCPT1/ ICPTCOL,*c_00k_* | 0.74673 | 0.55760 | 9 | 24.87888 | 0.003 |

#### Statistics for the current model

Deviance = 517.046336
Number of estimated parameters = 4

**Final model**

The maximum number of level-1 units = 120
The maximum number of row-level units = 93
The maximum number of column-level units = 10
The maximum number of iterations = 100

Method of estimation: full maximum likelihood
The maximum number of iterations = 100
Z-structure: independent

The outcome variable is REPORTIN 

### Summary of the model specified

#### Level-1 Model

*REPORTIN_ijk_* = *π_0jk_* + *π_1jk_**(*PUBLICAT_ijk_*) + *π_2jk_**(*JIF_ijk_*) + *e_ijk_*

#### Level-2 Model

*π_0jk_* = θ*_0_* + *b_00j_* + *c_00k_*
    *π_1jk_* = θ*_1_*
    *π_2jk_* = θ*_2_*

#### Mixed Model

*REPORTIN_ijk_* = θ*_0_* + θ*_1_***PUBLICAT_ijk_* + θ*_2_***JIF_ijk_*+ *b_00j_* + *c_00k_* + *e_ijk_*

For starting values, data from 120 level-1, 93 row-level
and 10 column-level records were used

## Final Results - iteration 21

**Iterations stopped due to small change in likelihood function**


σ^2^ = 3.37731

τ_rows_

| INTRCPT1 |
| --- |
| ICPTROW,*b_00j_* |
| 0.67753 |

τ_columns_

| INTRCPT1 |
| --- |
| ICPTCOL,*c_00k_* |
| 0.31314 |

The value of the log-likelihood function at iteration 21 = -2.565631E+002

#### Final estimation of fixed effects:

| Fixed Effect | Coefficient | Standard error | *t*-ratio | Approx. *d.f.* | *p*-value |
| --- | --- | --- | --- | --- | --- |
| For INTRCPT1, *π_0_* | | | | | |
| INTERCEPT,θ*_0_* | -116.759534 | 185.510600 | -0.629 | 17 | 0.537 |
| For PUBLICAT, *π_1_* | | | | | |
| INTERCEPT,θ*_1_* | 0.061890 | 0.092268 | 0.671 | 17 | 0.511 |
| For JIF, *π_2_* | | | | | |
| INTERCEPT,θ*_2_* | 0.455924 | 0.255219 | 1.786 | 17 | 0.092 |

#### Final estimation of row and level-1 variance components:

| Random Effect | Standard  Deviation | Variance  Component | *d.f.* | χ^2^ | *p*-value |
| --- | --- | --- | --- | --- | --- |
| INTRCPT1/ ICPTROW,*b_00j_* | 0.82312 | 0.67753 | 92 | 103.76653 | 0.189 |
| level-1, *e* | 1.83775 | 3.37731 |  |  |  |

#### Final estimation of column level variance components:

| Random Effect | Standard  Deviation | Variance  Component | *d.f.* | χ^2^ | *p*-value |
| --- | --- | --- | --- | --- | --- |
| INTRCPT1/ ICPTCOL,*c_00k_* | 0.55959 | 0.31314 | 9 | 17.60330 | 0.040 |

#### Statistics for the current model

Deviance = 513.126248
Number of estimated parameters = 6

1. **Calculation of the ICC**

Based on the intercept only output:

ICC at the author level:

$$\rho=\frac{b_{00j}}{b_{00j}+ c_{00k}+e}= \frac{0.78566}{0.78566+0.55760+ 3.32263}=0.168$$

ICC at the journal level:

$$\rho=\frac{c_{00k}}{b_{00j}+ c_{00k}+e}= \frac{0.55760}{0.78566+0.55760+ 3.32263}=0.120$$

Together the author and journal level account for 0.288 of the total variance:

$$\rho=\frac{b_{00j}+c_{00k}}{b_{00j}+ c_{00k}+e}= \frac{0.78566+0.55760}{0.78566+0.55760+ 3.32263}=0.288$$
